# Association between Vitamin D Deficiency and Serologic Response to Hepatitis B Virus Vaccination among Heavy Industry Workers

**DOI:** 10.3390/vaccines12070723

**Published:** 2024-06-28

**Authors:** Si-Ho Kim, Chang-Ho Chae

**Affiliations:** 1 Division of Infectious Diseases, Samsung Changwon Hospital, Sungkyunkwan University School of Medicine, Changwon 51353, Republic of Korea; 2 Department of Occupational and Environmental Medicine, Samsung Changwon Hospital, Sungkyunkwan University School of Medicine, Changwon 51353, Republic of Korea

**Keywords:** hepatitis B, hepatitis B vaccines, vitamin D, vitamin D deficiency, immunogenicity, vaccine

## Abstract

Introduction. Hepatitis B virus (HBV) vaccination has decreased the overall incidence of HBV infection; however, approximately 5 to 10% of people are non-responders to the vaccination. This study investigated the factors associated with non-response to HBV vaccination, with an emphasis on vitamin D deficiency (VDD). Methods. This retrospective observational study focused on adult workers in a single heavy industry. Individuals with negative initial hepatitis B surface antibody (anti-HBs) levels prior to vaccination and who then received a two- or three-dose series of HBV vaccinations were enrolled. The study endpoint was failure to achieve a seroprotective antibody response, defined as an anti-HBs titer less than 10 mIU/mL. Propensity score matching (PSM) and binary logistic regression models were used to adjust the outcomes for other clinical characteristics. Results. Among 760 workers, 566 (74.5%) exhibited VDD. The non-response rates to HBV vaccination were 13.4% (76/566) and 5.7% (11/194) among workers with and without VDD, respectively (*p* = 0.005). Even after adjustment using PSM, VDD was still associated with a higher rate of response failure (adjusted odds ratio 2.74; 95% confidence interval 1.40–5.38, *p* = 0.003). The binary logistic regression model showed that VDD, older age, omission of the third vaccine dose, lower initial anti-HBs titer, and current smoking were associated with response failure. Conclusions. Our study suggests that VDD may impair the serologic response following HBV vaccination. Further research is needed to evaluate the effectiveness of vitamin D supplementation in increasing the response to HBV vaccination.

## 1. Introduction

Infection with the hepatitis B virus (HBV) can cause chronic hepatitis, which can lead to liver cirrhosis and hepatocellular carcinoma [1]. The World Health Organization estimated that 254 million individuals worldwide had chronic hepatitis B in 2022, with 1.2 million patients newly diagnosed [2]. To prevent HBV transmission, a vaccine was developed, and second-generation DNA recombinant vaccines have become commonly adopted for universal vaccination [3]. The overall response rate to HBV vaccination among immunocompetent patients is 90–95%, although factors such as sex, age, smoking, and underlying diseases can affect the immune response [4].

In the Republic of Korea, the seroprevalence of the hepatitis B surface antigen (HBsAg) in the general population was higher than 8% before the launch of the national vaccine program in 1995. Since then, the seroprevalence of HBsAg has continuously decreased and was 2% in 2019 [5]. The program aims to vaccinate all newborns and provides three-dose of the hepatitis B vaccine (10 μg/0.5 mL per dose) free of charge. Hepatitis B is designated as a mandatory vaccination under the Korean Infectious Disease Prevention and Control Act [5,6]. Individuals who complete the three-dose vaccination are considered fully vaccinated, and it is not recommended to perform an anti-HBs test after vaccination in healthy individuals [7].

Meanwhile, although vitamin D is known to affect homeostasis between bone and minerals, there has recently been increased interest in its immunologic roles [8]. Vitamin D can inhibit B and T cell proliferation, shift T cell phenotypes toward weaker inflammatory types, and reduce inflammatory cytokine production, while enhancing levels of anti-inflammatory cytokines [8,9]. In addition, several in vitro and animal studies have suggested that vitamin D can influence vaccine responses via the interaction of 1,25-dihydroxyvitamin D with antigen presentation, as well as through its effects on dendritic cell migration and the subsequent activation of T and B cell antibody responses [9,10,11]. However, clinical studies have reported conflicting results regarding the association between the immune response to HBV vaccination and vitamin D [12,13,14,15]. A study of patients undergoing hemodialysis showed that VDD was associated with a decreased serologic response to HBV vaccination [12]; however, studies of healthy children did not show such an association [13]. Additionally, no randomized clinical trial has demonstrated the effect of vitamin D as a booster for HBV vaccine response [14,15].

The prevalence of VDD is common worldwide. Estimates of VDD prevalence were up to 40% in Europe, based on data from 14 nationally representative nutrition and health surveys conducted between 2002 and 2012 [16]. The Korea National Health and Nutrition Examination Survey data from 2008 to 2014 showed that 65.7% of males and 76.7% of females had VDD [17]. Therefore, VDD is common worldwide, including in developed countries, and can significantly affect public health. In this study, we evaluated the association between serum vitamin D concentration and serologic response to HBV vaccination in adults.

## 2. Methods

### 2.1. Study Site and HBV Vaccination Policy

This retrospective observational study was based on data from regular checkups and the HBV vaccination program at Samsung Heavy Industries in Geoje, conducted from December 2015 to September 2023. This company operates an HBV vaccination program for individuals with negative or trace results for hepatitis B surface antibody (anti-HBs). For this program, 1.0 mL of Euvax-B^®^ (produced by LG Chemical Ltd., Seoul, Republic of Korea) was administered. Additionally, all workers were subject to annual regular checkups, which included a laboratory test for anti-HBs and serum 25-OH vitamin D. The serum 25-OH vitamin D level was tested on the day of the first vaccination. All vaccinations and off-site annual checkups were administered by medical staff at Samsung Changwon Hospital. Anti-HBs and Vitamin D levels were measured by using a Cobas 8000 ^®^ auto-analyzer (Roche Diagnostics, Mannheim, Germany). The Elecsys Anti-HBs II system’s anti-HBs detection is reported by the manufacturer to have 100% sensitivity and over 99% specificity [18].

### 2.2. Study Eligibility Criteria

The inclusion criteria for this study were as follows: (1) adult workers (aged 18 or older) who had received a two-dose or three-dose series of the HBV vaccine during the vaccination program without documentation of a prior HBV vaccination and (2) adult workers with negative HBsAg. Because we hypothesized that there might be a difference in response between the patients receiving the two-dose or three-dose series of HBV vaccination stratified by VDD, we included both individuals receiving two or three-dose series of HBV vaccine. The exclusion criteria were workers without anti-HBs or serum 25-OH vitamin D data prior to vaccination and those with anti-HBs levels of 10 mIU/mL or higher.

### 2.3. Study Design, Definition, and Outcomes

In this study, we classified workers into two groups—those with VDD and those without VDD. VDD was defined as a serum 25-OH vitamin D level below 20 ng/mL, measured on the day of the first vaccination. This cut-off value was chosen considering that subjects with 25(OH)D levels of greater than 20 ng/mL did not experience significant changes in their PTH levels, and it is the most commonly adopted cut-off value [19]. We then compared the anti-HBs response between the two groups within three years following the HBV vaccination. If anti-HBs were measured more than twice, the highest level was selected as the study outcome. We also included the timing of selected anti-HBs measurements (when they peaked) as a variable in the analysis.

The primary endpoint of this study was the failure to achieve a seroprotective antibody response, defined as an anti-HBs level of less than 10 mIU/mL. The secondary endpoint was the anti-HBs titer after log transformation, with a measurable range of 2 to 1000 mIU/mL. In addition to serum 25-OH vitamin D level, the following patient demographics were collected from electronic medical records: age, sex, initial anti-HBs titer, body mass index, current smoking, hemoglobin A1 c, and underlying diseases (metabolic syndrome, diabetes mellitus, and chronic kidney disease ≥ stage 3). Given that the Korean National HBV vaccination program commenced in 1995, we included a birth year of 1995 or later as a variable potentially associated with vaccine response. This consideration stems from the likelihood that individuals born in 1995 or thereafter are more likely to have received the HBV vaccination, even if their anti-HBs levels were confirmed to be negative [5]. In addition, we also conducted an additional analysis to explore the risk factors for response failure in the overall study population and specifically among patients who completed a three-dose series of the vaccination.

### 2.4. Statistical Analysis

Statistical analyses were conducted using R software (Version 4.2.1; R Foundation for Statistical Computing, Vienna, Austria). To achieve covariate balance between groups, we utilized the MatchIt package for propensity score matching. Additionally, we employed the cobalt package to assess the balance of covariates before and after matching. To compare baseline characteristics between workers with and without VDD, Student’s *t*-test or the Mann–Whitney test for continuous variables were employed. Student’s *t*-test was used to compare the sizes of two independent groups following a normal distribution, while the Mann–Whitney test was used to compare the sizes of two groups that do not follow a normal distribution. The Chi-squared test or Fisher’s exact test for categorical variables were also used. The Chi-squared test was used when the cells with expected frequencies of less than 5 were 20% or fewer of the total, while Fisher’s exact test was used when they were more than 20%.

To adjust for the association between serologic response to vaccination and VDD, we used two adjustment models as follows: (1) a propensity score (PS) model with a matching weight of 1:2 and (2) a binary logistic regression model. For PS matching, propensity scores were calculated using logistic regression, incorporating variables such as age, BMI, timing of selected Anti-HBs measurement, current smoking, and hemoglobin A1c percentage. Non-matched cases were discarded. Covariate balance after weighting was assessed using the absolute standardized mean difference, with a standard difference of ≥0.10 considered a meaningful imbalance. In the binary logistic regression model, variables with a *p*-value < 0.20 in univariate analysis were included in the multivariable analysis. If variables were interrelated, we selected the one with the lowest *p*-value. We used the Hosmer–Lemeshow statistic to evaluate the goodness-of-fit of the final model. All *p*-values were two-tailed, and values < 0.05 were considered statistically significant.

### 2.5. Institutional Review Board Statement

The study was approved by the Institutional Review Board of Samsung Changwon Medical Center with a waiver for informed consent (IRB file number: SCMC 2024-04-019). The need for informed consent was waived because this was an observational retrospective study, and all patient data were analyzed anonymously.

## 3. Results

### 3.1. Study Population

**Figure 1:**
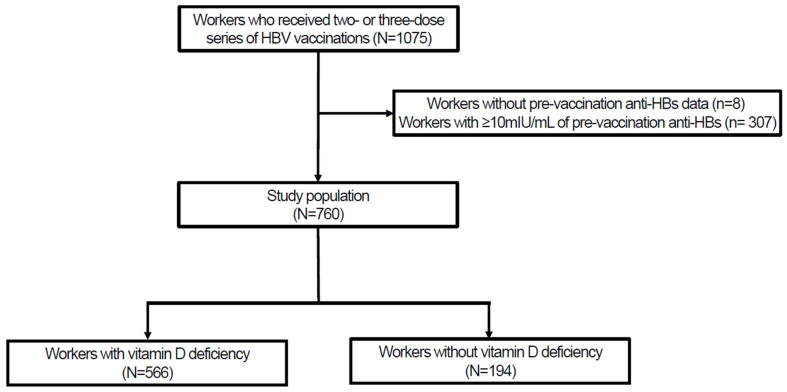
Study population; HBV, hepatitis B virus; Anti-HBs, hepatitis B surface antibody.

A total of 760 workers met the inclusion criteria (Figure 1). Among them, 566 (74.5%) had VDD, and the mean serum 25-OH vitamin D concentrations in patients with or without VDD were 13.87 ng/mL and 25.30 ng/mL, respectively (*p* < 0.001). There was a total of 1305 anti-HBs measurements in 760 workers, and the selected measurement points of anti-HBs tended to differ between workers with and without VDD, although the difference was not statistically significant. A total of 168 workers (22.1%) did not receive the third dose of the vaccine. When comparing the demographic data between the workers with or without VDD, those with VDD were younger. In addition, workers with VDD tended to have a lower body mass index and hemoglobin A1c level, although these differences were not statistically significant. No worker in either group had chronic kidney disease ≥ stage 3 (Table 1).

### 3.2. Immune Response after HBV Vaccination

**Figure 2:**
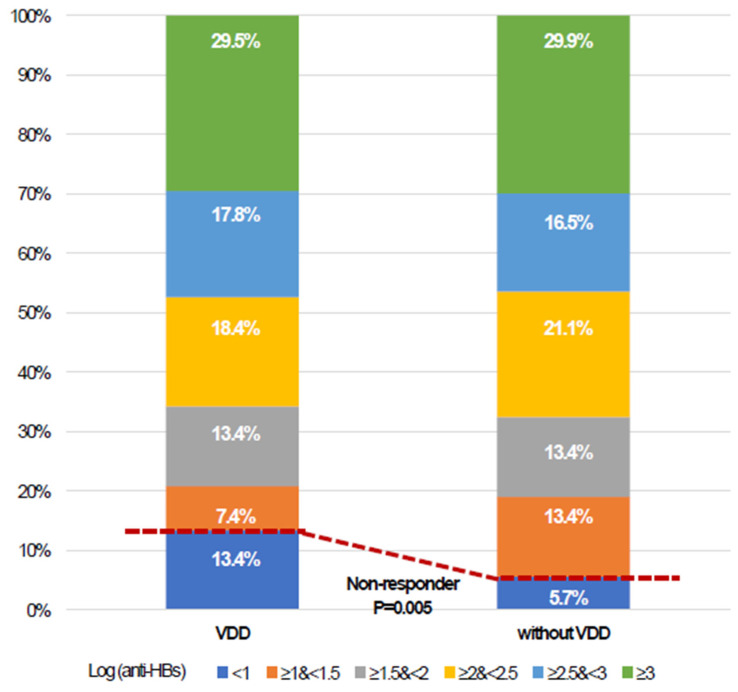
Distribution of serum hepatitis B surface antibody titers stratified by log-transformed values; VDD, vitamin D deficiency; Anti-HBs, hepatitis B surface antibody.

A total of 87 (11.4%) workers were classified as non-responders. The rates of response failure for workers with and without VDD were 13.4% and 5.7%, respectively (*p* = 0.005, Figure 2 and Table 2). The rate of response failure was higher in workers with VDD in both the two-dose series (25.0% vs. 13.6%, *p* = 0.118) and the three-dose group (10.2% vs. 3.3%, *p* = 0.009). After PS matching, the absolute standardized mean differences for all variables between the two groups were less than 0.1 in the adjusted model (Table 1). VDD was still associated with response failure even after adjustment (adjusted odds ratio [OR] 2.74, 95% confidence interval [CI] 1.40–5.38, *p* = 0.003, Table 2). However, there was no difference in the anti-HBs titer value with log transformation between the two groups after vaccination, either before or after adjustment (Figure 2 and Table 2). In the binary regression model for response failure in the overall population, VDD (Adjusted OR 3.20, 95% CI 1.60–6.38, *p* = 0.001) was consistently associated with response failure. Additionally, older age (Adjusted OR 1.06 per year, 95% CI 1.01–1.13, *p* = 0.038), a two-dose series of HBV vaccination (Adjusted OR 3.22, 95% CI 1.93–5.37, *p* < 0.001), and current smoking (Adjusted OR 1.83, 95% CI 1.12–2.99, *p* = 0.015) were associated with response failure. Conversely, a higher anti-HBs titer (Adjusted OR 0.47 per 1 mIU/mL 95% CI 0.33–0.65, *p* < 0.001) prior to vaccination was associated with a decreased risk of response failure. There was no association between a birth year of 1995 or later and response failure after HBV vaccination (Table 3). In the binary regression for response failure among patients who completed a three-dose series of vaccination, VDD (Adjusted OR 3.90, 95% CI 1.46–10.41, *p* = 0.007) was still associated with response failure (Table 4).

## 4. Discussion

Our study findings suggest that VDD is common in young individuals without chronic kidney disease in Republic of Korea and may influence serologic responses following HBV vaccination, along with factors such as age, the frequency of administration, current smoking, and pre-vaccination anti-HBs titer, even when the titer is less than 10 mIU/mL.

Numerous studies over the decades have evaluated the associations between various vaccines and immune responses [20]. Although more research is needed to understand the influence of vitamin D on humoral immunity mediated by B cells compared to innate or T cell immunity, it is hypothesized that the role of vitamin D in humoral immunity is associated with immune homeostasis and its immunomodulatory effects [8,20]. In addition, in vivo studies using animal models have suggested that the immune response to inactivated poliovirus vaccine and cold-attenuated influenza virus vaccine could be enhanced by administering vitamin D concurrent with vaccination [21,22]. However, Youssry et al. reported that vitamin D supplementation or ultraviolet B (UVB) exposure could have dual effects on HBV vaccine response. In their study, three groups of rabbits were classified as follows: (1) control, (2) rabbits exposed to UVB, and (3) rabbits that received commercial oral vitamin D. Serum vitamin D concentration was positively associated with serum anti-HBs levels within each group; however, mean anti-HBs IgG levels and avidity percentages were lower in the rabbits exposed to UVB and those that received oral vitamin D supplementation than in the control group [10].

Serial clinical studies have reported discrepant results regarding the association between vitamin D and immune responses following vaccination [20]. Because there has been great interest in the role of vitamin D in preventing viral respiratory illnesses, several prospective or randomized clinical trials have evaluated the immune response to vaccination according to vitamin D status or supplementation [23]. One RCT evaluating influenza vaccination observed higher plasma TGF-β concentrations after vaccination in volunteers with VDD after vitamin D supplementation; however, no corresponding increase in antibody response was noted [24]. Another RCT evaluating COVID-19 vaccination in volunteer women with or without vitamin D supplementation showed a significantly positive correlation between immunoglobulin-G and 25-OH vitamin D levels [25]. However, the largest prospective study in adults aged ≥ 50 years found no consistent correlation between vitamin D status and serologic response after influenza vaccination [26].

With regard to HBV vaccination, one retrospective analysis of patients with chronic kidney disease stages 3–5 revealed that those with VDD had lower seroconversion rates than those without VDD (45% vs. 64%; *p* = 0.011) [12]. However, a randomized open-label pilot study involving hemodialysis patients showed that oral vitamin D supplementation did not improve serologic response after HBV vaccination, although the study involved a relatively small number of participants (20 patients in the supplementation group and 17 in the control group) [15]. Notably, about 50% of the patients in the vitamin D supplementation group did not achieve a serum 25-OH vitamin D concentration of 30 ng/mL, which was the pre-specified definition of VDD in this study. Additionally, a placebo-controlled study with volunteers from the British Army who received oral vitamin D and simulated sunlight indicated that an initial low vitamin D status was associated with a poor vaccine response. However, even with supplementation, there was no improvement in vaccine response [14]. In this placebo-controlled study, vitamin D supplementation was initiated after the first HBV vaccination. Therefore, there was concern about ensuring an appropriate serum vitamin D level before initiating HBV vaccination. Meanwhile, a retrospective study of healthy children younger than 12 years showed that age—the time interval from the last dose of the HBV vaccination—was the only factor associated with a decline in anti-HBs titer. Vitamin D status had no impact on anti-HBs status [13]. In our study, the seroprotective response rate after HBV vaccination was higher in patients without VDD than in those with VDD; however, there was no association between serum 25-OH vitamin D level and anti-HBs titer. The reasons for these discrepancies are unknown; however, it might be that the immunologic role of vitamin D is more strongly related to immune modulation than immune augmentation [8]. Therefore, it can be assumed that, even though seroconversion was frequently observed in workers without VDD, the overall anti-HBs titers of workers with or without VDD did not differ. Additionally, prior studies have shown that simple vitamin D supplementation is not associated with an increased immune response; therefore, an appropriate dose and timely administration of vitamin D supplementation might be necessary to enhance the serologic response effects of vitamin D. Additionally, because vitamin D is not a panacea and its supplementation is most effective only in patients with VDD, these supplementation studies should be focused on patients with VDD and aim to identify the groups most responsive to VDD supplementation [27].

In our study, a higher anti-HBs titer before vaccination was associated with an appropriate serologic response after HBV vaccination, along with age and completion of the three-dose series. To the best of our knowledge, there are no data on the relationship between anti-HBs titer prior to vaccination and seroprotective response among individuals without documented HBV vaccination. However, a study from Italy involving vaccinated university students showed that students with a detectable but negative anti-HBs titer (2.00–9.99 mIU/mL) before booster vaccination had a seroprotective response after a booster vaccination compared to those with a negative and non-detectable anti-HBs titer (<2.00 mIU/mL) [28]. It is known that anti-HBs levels wane over time; however, it is also known that more than 80% of vaccinated individuals with negative anti-HBs show an appropriate anti-HBs response after booster vaccination, known as the anamnestic anti-HBs response [29,30]. Therefore, booster vaccinations are not recommended for immunocompetent individuals, whether they have negative or positive anti-HBs, if they have been appropriately vaccinated [31]. However, based on our study and the one conducted in Italy, individuals with documented vaccination but lower or non-detectable anti-HBs levels within the negative range appear to have a diminished anamnestic response and might benefit more from alternative HBV prevention strategies than from booster vaccinations [28]. Similarly, individuals without a documented vaccination have a higher probability of response failure; therefore, alternative HBV vaccination or prevention strategies may be necessary for these individuals.

Our study has several limitations. First, as this study was conducted at a single company with high proportions of male and young workers with VDD, application of our results to other populations must be performed with caution. Second, due to the inherent limitations of an observational study design, we were not able to obtain data related to vitamin D supplementation. However, no randomized controlled trial has convincingly demonstrated a link between vitamin D supplementation and improved HBV vaccine response [14,15]. Therefore, the usual dose of vitamin D supplementation might not have a significant effect on the serologic response. In addition, about one-fifth of the workers in this study received only two doses of the vaccination. Due to the inherent limitations of an observational study, we could not confirm the reason why these workers did not receive the third dose of the vaccine. However, since this vaccination program was voluntary, the completeness of vaccination might be lower than in national mandatory vaccination programs. Despite this, the effect of VDD on the serologic response remained consistently low in patients receiving either the two-dose or three-dose series of the HBV vaccine. Third, individuals with VDD and those without VDD have different characteristics. Although we conducted PS matching to reduce bias, differences in clinical characteristics that could affect the serologic response to the HBV vaccine might still remain. Additionally, there might be unmeasured variables that may influence serologic response after HBV vaccination. Fourth, we only evaluated serologic responses after HBV vaccination. It is known that even vaccinated individuals with negative serum anti-HBs can show T cell proliferative responses and HBV-reactive CD4 T cells [32,33]; however, further studies are needed to evaluate the association between VDD and T cell responses after HBV vaccination.

## 5. Conclusions

Our study suggests that vitamin D plays an important role in the seroprotective response following HBV vaccination among young healthy workers without chronic kidney disease. Additionally, the anti-HBs titer is also important for predicting serologic responses, even in individuals with a negative range of anti-HBs titer. Given the conflicting results and limitations of current research on vitamin D supplementation and HBV vaccination, further prospective studies are needed to evaluate various and unique approaches to vitamin D supplementation. These studies should ensure a sufficient time interval between the initiation of vitamin D supplementation and HBV vaccination to secure appropriate serum vitamin D levels at the time of the first vaccination.

## Figures and Tables

**Table 1:** Comparison of demographics among patients with and without vitamin D deficiency.

				Unadjusted			After PS Matching (1:2)		
	Overall (760)	Without Vit. D Deficiency (194)	With Vit. D Deficiency (566)	*p*-Value	ASMD	Without Vit. D Deficiency (194)	With Vit. D Deficiency (388)	*p*-Value	ASMD
Serum 25-OH Vitamin D (ng/mL)	16.79 ± 6.62, 15.97 (12.35–20.16)	25.30 ± 5.75, 24.29 (22.39–27.40)	13.87 ± 3.76, 14.26 (11.29–16.99)	<0.001	2.350	25.30 ± 5.75, 23.24 (21.65–27.01)	13.99 ± 3.77, 14.47 (11.29–17.17)	<0.001	2.323
Age (years)	29.74 ± 4.40, 29 (26–33)	30.60 ± 4.39, 30 (27–34)	29.44 ± 4.37, 29 (26–33)	0.002	0.265	30.60 ± 4.39, 30 (27–34)	30.33 ± 4.18, 30 (27–34)	0.449	0.063
Birth year 1995 or later	24 (3.2)	5 (2.6)	19 (3.4)	0.592	0.046	5 (2.6)	9 (2.3)	>0.999	0.017
Sex (male)	687 (90.4)	178 (91.8)	509 (89.9)	0.572	0.063	178 (91.8)	348 (89.7)	0.426	0.070
Anti-HBs titer prior to vaccination (mIU/mL)	3.44 ± 2.18, 2 (2–4.49)	3.45 ± 2.24, 2 (2–4.86)	3.43 ± 2.16, 2 (2–4.36)	0.929	0.007	3.45 ± 2.24, 2 (2–4.86)	3.50 ± 2.22, 2 (2–4.51)	0.330	0.021
Received two doses of the HBV vaccination	168 (22.1)	44 (22.7)	124 (21.9)	0.841	0.019	44 (22.7)	83 (21.4)	0.723	0.031
Timing of selected Anti-HBs measurement				0.053	0.202			0.940	0.032
The following year after vaccination	476 (62.6)	134 (69.1)	342 (60.4)			134 (69.1)	263 (67.8)		
Two years after vaccination	271 (35.7)	59 (30.4)	212 (37.5)			59 (30.4)	122 (31.4)		
Three years after vaccination	13 (1.7)	1 (0.5)	12 (2.1)			1 (0.5)	3 (0.8)		
Body mass index (kg/m^2^)	24.59 ± 3.55, 24.21 (22.13–26.78)	25.01 ± 3.60, 24.29 (22.39–27.40)	24.45 ± 3.51, 24.16 (21.93–26.59)	0.060	0.156	25.01 ± 3.60, 24.29 (22.39–27.40)	24.48 ± 3.54, 24.48 (22.35–26.88)	0.747	0.049
Current smoking	267 (35.1)	58 (29.9)	209 (36.9)	0.077	0.149	58 (29.9)	120 (30.9)	0.799	0.022
Hemoglobin A1c (%)	5.34 ± 0.54, 5.3 (5.1–5.5)	5.40 ± 0.68, 5.3 (5.1–5.5)	5.32 ± 0.48, 5.3 (5.1–5.5)	0.073	0.136	5.40 ± 0.68, 5.3 (5.1–5.5)	5.36 ± 0.55, 5.3 (5.2–5.5)		0.064
Presence of diabetes mellitus				0.179	0.121			0.687	0.032
Normal	683 (89.9)	168 (86.6)	515 (91.0)			168 (86.6)	343 (88.4)		
Prediabetes	66 (8.7)	23 (11.9)	43 (7.6)			23 (11.9)	38 (9.5)		
Diabetes mellitus	11 (1.4)	3 (1.5)	8 (1.4)			3 (1.5)	8 (1.9)		
Metabolic syndrome	62 (8.2)	20 (10.3)	42 (7.4)	0.205	0.102	20 (10.3)	54 (9.3)	0.521	0.053
Chronic kidney disease ≥ stage 3	0 (0.0)	0 (0.0)	0 (0.0)	>0.999	N/A	0 (0.0)	0 (0.0)	>0.999	N/A

Values are presented as mean ± standard deviation, median (interquartile range), or number (%). Because the continuous variables did not follow a normal distribution, the Mann–Whitney test was used to compare the sizes of the two groups. Anti-HBs, hepatitis B surface antibody; HBV, hepatitis B virus; ASMD, absolute standardized mean difference.

**Table 2:** Comparison of hepatitis B vaccination response among patients with and without vitamin D deficiency.

				Non-Matched			PS-Matched		
	Overall (760)	Without Vit. D Deficiency (194)	With Vit. D Deficiency (566)	OR (95% CI)	*p*-Value	Without Vit. D Deficiency (194)	With Vit. D Deficiency (388)	OR (95% CI)	*p*-Value
Primary outcome									
Non-responder	87 (11.4)	11 (5.7)	76 (13.4)	2.58 (1.34–4.97)	0.005	11 (5.7)	55 (14.2)	2.74 (1.40–5.38)	0.003
Secondary outcome									
Anti-HBs response (1og transformation, mIU/mL)	2.19 ± 0.84, 2.41 (1.70–3.00)	2.25 ± 0.75, 2.43 (1.70–3.00)	2.18 ± 0.87, 2.41 (1.70–3.00)	N/A	0.684	2.25 ± 0.75, 2.43 (1.70–3.00)	2.15 ± 0.88, 2.39 (1.66–3.00)	N/A	0.494

Values are presented as mean ± standard deviation, median (interquartile range), or number (%). Anti-HBs, hepatitis B surface antibody.

**Table 3:** Factors associated with HBV response failure.

	Immune Response		Univariate		Binary Logistic Model	
	Non-Responder	Responder	OR (95% CI)	*p*-Value	OR (95% CI)	*p*-Value
Age (per year)	30.49 ± 4.58, 31 (27–34)	29.64 ± 4.37, 29 (26–33)	1.05 (0.99–1.10)	0.090 *	1.06 (1.01–1.13)	0.038
Birth year ≥ 1995	5 (20.8)	19 (79.2)	2.10 (0.76–5.77)	0.151 *	1.59 (0.48–1.13)	0.450
Sex (male)	83 (12.1)	604 (87.9)	2.37 (0.84–6.67)	0.102 *	1.83 (0.62–5.47)	0.276
Anti-HBs titer prior to vaccination (per 1 mIU/mL)	2.21 ± 0.73, 2 (2–2)	3.60 ± 2.26, 2 (2–4.93)	0.49 (0.35–0.67)	<0.001 *	0.47 (0.33–0.65)	<0.001
Received two doses of the HBV vaccination	37 (22.0)	131 (78.0)	3.06 (1.92–4.88)	<0.001 *	3.22 (1.93–5.37)	<0.001
Timing of Anti-HBs measurement				0.314		
The following year after vaccination	49 (10.3)	427 (89.7)	Reference			
Two years after vaccination	38 (14.0)	233 (86.0)	1.42 (0.90–2.24)			
Three years after vaccination	0 (0.0)	13 (100.0)	N/A			
Vitamin D deficiency	76 (13.4)	490 (86.6)	2.58 (1.34–4.97)	0.005 *	3.20 (1.60–6.38)	0.001
Body mass index (per 1 kg/m^2^)	24.81 ± 3.46, 24.22 (22.25–27.15)	24.56 ± 3.56, 24.20 (22.10–26.61)	1.02 (0.96–1.08)	0.550		
Current smoking	41 (15.4)	226 (84.6)	1.76 (1.12–2.77)	0.014 *	1.83 (1.12–2.99)	0.015
Hemoglobin A1c (per 1%)	5.39 ± 0.58, 5.3 (5.2–5.5)	5.33 ± 0.53, 5.3 (5.1–5.5)	1.16 (0.83–1.62)	0.373		
Presence of diabetes mellitus				0.155		
Normal	73 (10.7)	610 (89.3)	Reference			
Prediabetes	12 (18.2)	54 (81.8)	1.86 (0.95–3.63)			
Diabetes mellitus	2 (18.2)	9 (81.8)	1.86 (0.39–8.76)			
Metabolic syndrome	11 (17.7)	51 (82.3)	1.77 (0.88–3.53)	0.108 *	2.07 (0.95–4.53)	0.068

Values are presented as mean ± standard deviation, median (interquartile range), or number of non-responders and responders (%). Anti-HBs, hepatitis B surface antibody; HBV, hepatitis B virus. * Variables included in the binary logistic regression analysis. Hosmer–Lemeshow test *p* = 0.810.

**Table 4:** Factors associated with HBV vaccine response failure among patients who completed a three-dose series of vaccination.

	Immune Response		Univariate		Binary Logistic Model	
	Non-Responder (50)	Responder (542)	OR (95% CI)	*p*-Value	OR (95% CI)	*p*-Value
Age (per year)	30.88 ± 4.53 31.5 (28–35)	29.66 ± 4.27, 29 (26–33)	1.07 (1.00–1.14)	0.057 *	1.11 (1.03–1.20)	0.008
Birth year ≥ 1995	3 (27.3)	8 (72.7)	4.26 (1.09–16.6)	0.037 *	5.02 (1.05–24.12)	0.044
Sex (male)	48 (9.0)	483 (91.0)	2.92 (0.70–12.38)	0.143 *	2.83 (0.63–12.82)	0.176
Anti-HBs titer prior to vaccination (per 1 mIU/mL)	2.25 ± 0.79 2 (2–2)	3.62 ± 2.27 2(2–4.94)	0.51 (0.34–0.75)	0.001 *	0.48 (0.31–0.73)	0.001
Timing of Anti-HBs measurement				0.447		
The following year after vaccination	28 (7.5)	346 (92.5)	Reference			
Two years after vaccination	22 (10.6)	186 (89.4)	1.46 (0.81–2.63)			
Three years after vaccination	0 (0.0)	10 (100.0)	N/A			
Vitamin D deficiency	45 (10.2)	397 (89.8)	3.29 (1.28–8.44)	0.013 *	3.90 (1.46–10.41)	0.007
Body mass index (per 1 kg/m^2^)	24.96 ± 3.49, 24.01 (22.27–27.14)	24.61 ± 3.61, 24.18 (22.16–26.60)	1.03 (0.95–1.11)	0.512		
Current smoking	30 (14.0)	185 (86.0)	2.90 (1.60–5.24)	<0.001 *	3.01 (1.61–5.62)	0.001
Hemoglobin A1c (per 1%)	5.38 ± 0.70, 5.4 (5.1–5.5)	5.34 ± 0.57 5.3 (5.1–5.5)	1.10 (0.73–1.67)	0.636		
Presence of diabetes mellitus				0.814		
Normal	44 (8.2)	492 (91.8)	Ref			
Prediabetes	5 (10.6)	42 (89.4)	1.33 (0.50–3.54)			
Diabetes mellitus	1 (11.1)	9 (88.9)	1.40 (0.17–11.43)			
Metabolic syndrome	6 (11.8)	45 (88.2)	1.51 (0.61–3.73)	0.376		

Values are presented as mean ± standard deviation, median (interquartile range), or number of non-responders and responders (%). Anti-HBs, hepatitis B surface antibody; HBV, hepatitis B virus. * Variables included in the binary logistic regression analysis. Hosmer–Lemeshow test *p* = 0.271.

## Data Availability

The raw data supporting the conclusions of this article will be made available by the authors on request.
